# Omalizumab in patients with severe asthma and persistent sputum eosinophilia

**DOI:** 10.1186/s13223-019-0337-2

**Published:** 2019-04-03

**Authors:** Manali Mukherjee, Melanie Kjarsgaard, Katherine Radford, Chynna Huang, Richard Leigh, Delbert R. Dorscheid, Catherine Lemiere, Louis-Philippe Boulet, Susan Waserman, James Martin, Parameswaran Nair

**Affiliations:** 1 Department of Medicine, Division of Respirology, McMaster University, Hamilton, ON Canada; 2 Firestone Institute for Respiratory Health, St Joseph’s Healthcare, 50 Charlton Avenue East, Hamilton, ON L8N 4A6 Canada; 3 Department of Medicine, University of Calgary, Calgary, Canada; 4 Department of Medicine, University of British Columbia, Vancouver, BC Canada; 5 Faculty of Medicine, Université de Montréal, Montreal, QC Canada; 6 Centre de recherche de l’Institut universitaire de cardiologie et de pneumologie de Québec (CRIUCPQ), Université Laval, Quebec, QC Canada; 7 Department of Medicine, Division of Allergy and Clinical Immunology, Faculty of Medicine, McGill University, Montreal, QC Canada; 8 Meakins-Christie Laboratories, Research Institute of the McGill University Health Centre, Montreal, Canada

**Keywords:** Severe asthma, Sputum eosinophils, Omalizumab, IgE, TSLP

## Abstract

Omalizumab, a recombinant humanized monoclonal antibody targeting the IgE molecule, is the first biologic approved for moderate-to-severe allergic asthmatics, who remain uncontrolled despite high dose inhaled corticosteroid and bronchodilators. Steroid-sparing effect of omalizumab has not been demonstrated in asthmatics with persistent airway eosinophilia in a randomised controlled trial till date. From this double-blind, placebo-controlled, multi-centred, randomized parallel group design, we report that omalizumab is possibly inadequate to control sputum eosinophilia, and therefore may not have a steroid-sparing effect, especially in those maintained on oral corticosteroids daily. This needs to be confirmed or refuted in a larger trial, which may be a challenge with respect to recruitment, since there are currently three additional biologics available to prescribe.

*Trial registration* Clinicaltrials.gov, NCT02049294, Registered 30th January 2014, https://clinicaltrials.gov/ct2/show/NCT02049294

**Electronic supplementary material:**

The online version of this article (10.1186/s13223-019-0337-2) contains supplementary material, which is available to authorized users.

To the editor:

Omalizumab, a monoclonal antibody (mAb) targeting circulating IgE, is the earliest approved mAb therapy in asthma. In asthmatics inadequately controlled despite high dose inhaled corticosteroid (ICS) and long-acting bronchodilator therapy, omalizumab decreased the level of free-circulating IgE, leading to improvements in patient quality of life, and decreased exacerbations with a 25% relative risk reduction compared to placebo (EXTRA study) [1]. Insufficient evidence of benefit has been found in participants specifically with severe oral corticosteroid (OCS)-dependent asthma [2]. Although the drug does enable a modest reduction in the dose of inhaled steroids (ICS), almost all the evidence has come from studies that evaluated patients who are on moderate to high doses of corticosteroids (< 1500 mcg daily of fluticasone equivalent). These studies did not rigorously establish the maintenance doses of corticosteroids by monitoring steroid-responsive biomarkers such as exhaled nitric oxide or sputum eosinophil counts that can be attenuated by omalizumab, at least in patients with mild to moderate asthma [3]. Therefore, in a double-blind, placebo-controlled, multi-centred, randomized parallel group design, we investigated whether omalizumab could control sputum eosinophilia that is not controlled by high doses of ICS (with or without OCS) in allergic asthmatics. In addition, we assessed whether omalizumab might allow a reduction in the dose of ICS or OCS in patients maintained on high doses of ICS and/or OCS without losing asthma control.

From six academic centres, we recruited 11 patients with confirmed asthma (12% bronchodilator reversibility or PC_20_ methacholine less than 8 mg/mL), atopy (skin prick test positive to common aeroallergens and elevated serum IgE levels), who were symptomatic (ACQ-5 ≥ 1.5) with evidence of sputum eosinophils (> 3%) despite high dose maintenance corticosteroid therapy. The trial was divided into two sequential study periods, where ‘phase 1’ saw randomisation (1:1) to either placebo or intervention for 16 weeks (either once monthly for 4 months or every 2 weeks for 4 months, dependent on the body weight and IgE level). Phase 2 involved a standardised corticosteroid reduction at intervals of 4 weeks while on the same intervention/placebo regime (week 16–week 32). A consort flow diagram has been provided in Fig. 1. Baseline characteristics of patients included in analysis for both drug and placebo arms were comparable (Table 1). Nine patients out of eleven randomised were included in the final analysis (two patients were excluded at physician’s discretion, refer to Fig. 1 consort flow diagram). Outcomes for both drug (n = 4) and placebo (n = 5) arms were analysed at week 32 Mann Whitney U test compared changes (week 32–week 0) with drug versus placebo, while in-group differences were compared using Wilcoxon analysis.

**Fig. 1:**
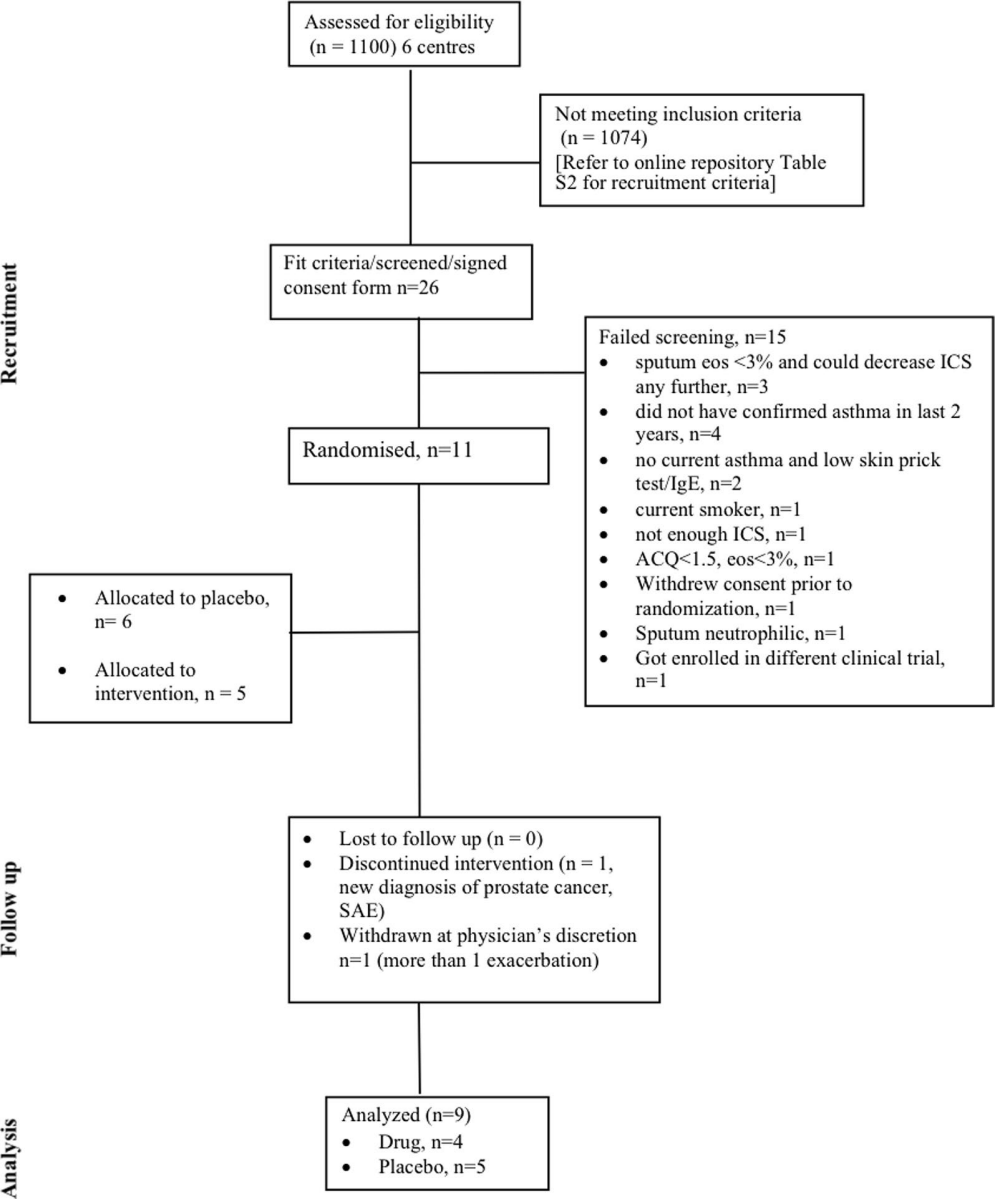
Consort flow diagram of patient recruitment: details of patient recruitment process, study and analysis is provided in a schematic

**Table 1:** Demographics of patients included in the analysis

Patient characteristics	Drug	Placebo	*P* value
	(n = 4)	(n = 5)	
Age (years)	54.5 ± 17.3	58.8 ± 9.5	0.64
Sex (F, n)	1	1	0.99
BMI	30.15 ± 5.1	28.8 ± 5.9	0.72
FEV_1_ %predicted	60 ± 15.5	51.6 ± 13.1	0.41
FEV_1_/VC	0.64 ± 0.15	0.56 ± 0.09	0.36
Serum IgE	965 ± 1499	482 ± 696	0.58
ACQ-5	1.79 ± 0.5	2.2 ± 0.67	0.35
Blood eosinophil (× 10^9^/L)	0.35 ± 0.23	0.62 ± 0.50	0.36
Sputum eosinophil (%)	15.75 ± 11.1	22.18 ± 14.02	0.5
Prednisone use (n)	1 (4)	0	0.29
Inhaled corticosteroid (median, max–min)	1450 (2000, 800)	1500 (2400, 1250)	0.37

N.B. data presented as mean ± standard deviation, unless otherwise mentioned. All patients were skin prick test positive

With respect to the primary outcome (reduction in sputum eosinophilia, Fig. 2a), omalizumab was unable to reduce sputum eosinophils (mean change in sputum eosinophils in drug arm = 2.65, placebo arm = 7.02, P = 0.6). In fact, at week 32 there was 16% increase in sputum eosinophils in the drug arm compared to 30% in the placebo arm (P = 0.7). With respect to blood eosinophils, there was no change in the absolute values in the drug arm (n = 4), while a placebo effect was apparent (9.6% decrease, P = 0.75, Fig. 2b).

**Fig. 2:**
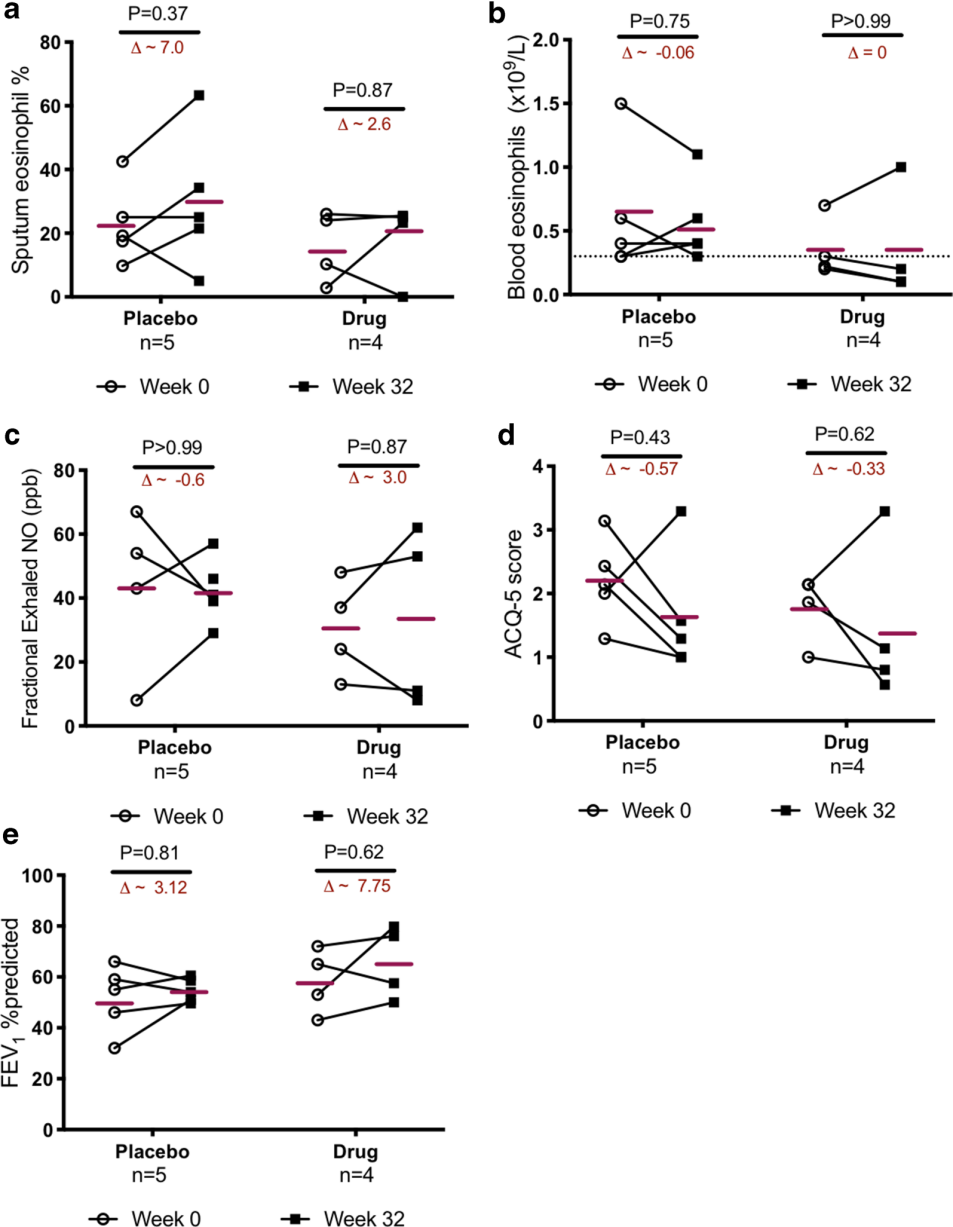
Effect of omalizumab on eosinophilia and indices of asthma severity: no significant reduction in eosinophils in the **a** sputum or **b** circulation, nor in the measurements of **c** fractional exhaled nitric oxide (FeNO), **d** asthma control (ACQ-5), or **e** FEV_1_ %predicted, could be documented for patients in the drug arm compared to placebo. Bars within the plots indicate the mean value for each group, and the delta (∆) values reflecting the mean absolute change within the group is give. Changes from baseline were compared for each arm using Wilcoxon paired analysis. P values were > 0.05, and were deemed non-significant

With respect to exacerbations, one patient in the drug arm, with a prednisone maintenance dose (at start of trial) of 12.5 mg daily exacerbated when dose was tapered to 5 mg. The exacerbation was treated with a prednisone burst (30 mg × 5 days) and patient was reinstated back on 12.5 mg. The remaining 3 patients in the drug arm reduced ICS dose (expressed as equivalent of fluticasone propionate) from a mean of 1400 mcg to 400 mcg (70%) while maintaining control. In the placebo arm, 4 out of 5 patients exacerbated on reduction of steroid dose (80%). All patients in the placebo arm were ICS-dependent. Only 1 could reduce ICS by 80% (maintaining control), while the remaining four exacerbated with decrease in ICS by 50%. Due to the small sample size, significance was not reached (with respect to reduction in corticosteroid therapy by 50% while maintaining asthma control). The steroid-sparing effect of omalizumab based on our observation warrants for further investigation. It is also important to note that the difference in daily exposure to perennial allergens between the recruited patients may also pose a factor that underlies responsiveness to an anti-IgE molecule. This factor could have been negated with a bigger sample size.

Similarly, there was no conclusive treatment effect observed in any of the secondary outcomes; for example, fractional exhaled nitric oxide (FeNO, Fig. 2c), asthma control questionnaire-5 (ACQ, Fig. 2d), lung function (FEV_1_, Fig. 2e), in neither the drug arm nor compared to placebo.

As an exploratory outcome, the eosinophil clonogenic potential was measured as eosinophil/basophil (Eo/B) colony forming units from peripheral blood mononuclear cells obtained from patients recruited in the trial, with written consent. We investigated whether in the event of IgE blockade, the effects of thymic stromal lymphopoietin (TSLP) on its described role of eosinophil recruitment and in situ eosinophilopoiesis [4] would be redundant. In addition, we also investigated whether the leukotriene antagonist montelukast, had similar effects, since the magnitude of clinical effect reported in the anti-TSLP clinical trial [5] was similar to those previously reported with montelukast [6]. Eo/B colonies enumerated at baseline, and at end of phase 1 (week 16) showed no significant difference between the respective drug and placebo arms (Additional file 1: Figure S1). At baseline (before any intervention), the clonogenic potential was comparable between the two arms for all tested conditions. Though insignificant, a trend was observed that showed synergistic effect of TSLP and IL-5 on eosinophilopoiesis as previously established (measured by the enumeration of Eo/B colonies, in vitro). At end of 16 weeks (Phase 1, referred to as Post-Rx in Additional file 1: Figure S1), there was again no comparable difference noticed, indicating that IgE blockade may not have a direct effect on the clonogenic potential.

We acknowledge that the sample size of this study is a major limitation. We calculated a sample size of 24 patients (12 in each arm) to give us 80% power to show a 50% decrease in the dose of maintenance glucocorticosteroids and a 50% reduction in sputum eosinophils [7]. As shown in the consort diagram (Fig. 1), patient recruitment for this trial was severely compromised due to two primary factors (i) potentially eligible patients having previously been prescribed Xolair^®^, and had failed to show expected clinical response, and (ii) the availability of anti-IL-5 biologics once the study had started (refer to Additional file 2: Table S1 for study exclusion criteria). As such we had only 40% power to draw our conclusions. In summary, we do not believe that omalizumab is able to reduce sputum eosinophilia in atopic asthmatics on high maintenance doses of glucocorticosteroids. A larger clinical trial is necessary to examine if it improves asthma control, reduces exacerbations and the maintenance dose of glucocorticosteroids by alternate mechanisms. Our experience also demonstrates the significant limitation of conducting clinical trials to evaluate biologics in academic centres in Canada in the current environment when four biologics (including omalizumab) are currently approved and commercially available.

## Additional files

**Additional file 1: Figure S1.** Clonogenic potential of TSLP in patients treated with Omalizumab: Eo/B colonies enumerated at baseline, and at end of phase 1 (week 16) showed no significant difference between the respective drug and placebo arms. Data is presented as mean (SD), for drug (n = 4) and placebo arm. (n = 4). One set of data from drug arm was excluded due to contamination in two of the colony plates. Two-way ANOVA was used for analysis. P values were deemed non-significant.

**Additional file 2: Table S1.** Criteria for patient recruitment.

## Abbreviations

mAb: monoclonal antibody
ACQ-5: asthma control questionnaire-5
IgE: immunoglobulin E
TSLP: thymic stromal lymphopoietin
Eo/B cfu: eosinophil/basophil colony forming units
FeNO: fractional exhaled nitric oxide
ICS: inhaled corticosteroid
OCS: oral corticosteroid
