# Supplementary figures and images for: Omalizumab in patients with severe asthma and persistent sputum eosinophilia
Source: Allergy Asthma Clin Immunol. 2019 Apr 3;15:21. doi: 10.1186/s13223-019-0337-2 (PMC6448265; doi:10.1186/s13223-019-0337-2)

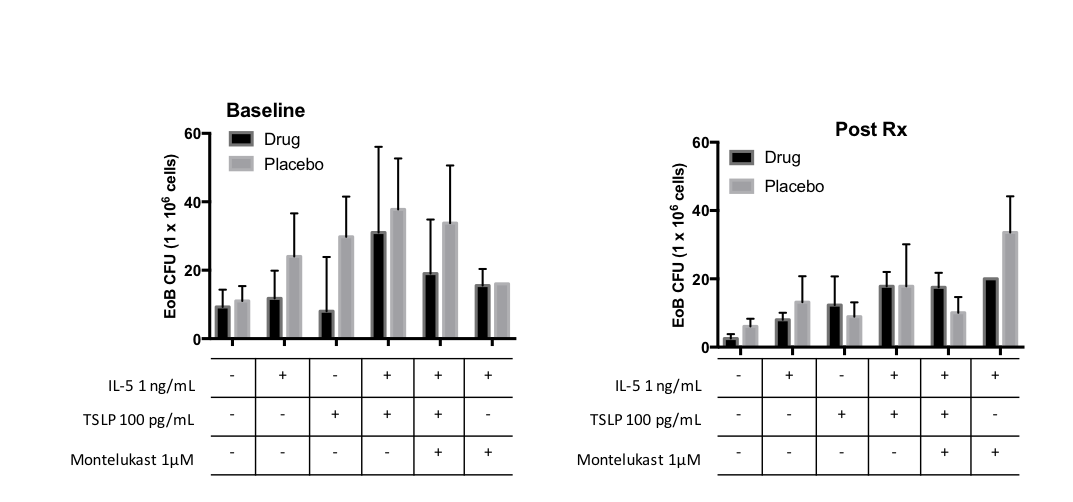

Supplement: Supplementary file 1 — Additional file 1: Figure S1. Clonogenic potential of TSLP in patients treated with Omalizumab: Eo/B colonies enumerated at baseline, and at end of phase 1 (week 16) showed no significant difference between the respective drug and placebo arms. Data is presented as mean (SD), for drug (n = 4) and placebo arm. (n = 4). One set of data from drug arm was excluded due to contamination in two of the colony plates. Two-way ANOVA was used for analysis. P values were deemed non-significant. [file 13223_2019_337_MOESM1_ESM.png]
