# Supplementary material for: Omalizumab in patients with severe asthma and persistent sputum eosinophilia
Source: Allergy Asthma Clin Immunol. 2019 Apr 3;15:21. doi: 10.1186/s13223-019-0337-2 (PMC6448265; doi:10.1186/s13223-019-0337-2)
Supplement: Supplementary file 2 — Additional file 2: Table S1. Criteria for patient recruitment. [file 13223_2019_337_MOESM2_ESM.docx]

*Omalizumab in severe eosinophilic asthma*

*Online Repository*

**Table S1: Criteria for patient recruitment**

| **Inclusion Criteria** | **Exclusion Criteria** |
| --- | --- |
| 1. Confirmed asthma within the past 2 years (12% bronchodilator reversibility or PC20 methacholine less than 8 mg/ml) 2. ACQ ≥1.5 and sputum eos ≥3% at the time of randomization 3. On ICS (≥ 1500 mcg fluticasone propionate or equivalent) with or without additional prednisone 4. Total serum IgE ≥30 IU/L and positive allergy skin prick test 5. Age between 18 and 75 years 6. Ability to provide informed consent | 1. Current smoker or ex-smokers with greater than 20 pack years  2. Co-morbid diseases which in the investigator's opinion would make the patient unsuitable to participate in the study  3. Currently on Omalizumab or has previously been treated with Omalizumab  4. Currently on other biologic therapies (eg. Prolia)  5. Pregnancy or lactation  6. Post bronchodilator FEV1 less than 50% predicted |
